# Consequences of Aging on Bone

**DOI:** 10.14336/AD.2023.1115

**Published:** 2023-11-20

**Authors:** Lingli Zhang, Qiao Guan, Zhikun Wang, Jie Feng, Jun Zou, Bo Gao

**Affiliations:** ^1^College of Athletic Performance, Shanghai University of Sport, Shanghai, China; ^2^School of Exercise and Health, Shanghai University of Sport, Shanghai, China; ^3^Department of Orthopedic Surgery, Xijing Hospital, Air Force Medical University, Xi’an, China

**Keywords:** aging, bone microenvironment, bone cells, bone immunity, osteoporosis

## Abstract

With the aging of the global population, the incidence of musculoskeletal diseases has been increasing, seriously affecting people's health. As people age, the microenvironment within skeleton favors bone resorption and inhibits bone formation, accompanied by bone marrow fat accumulation and multiple cellular senescence. Specifically, skeletal stem/stromal cells (SSCs) during aging tend to undergo adipogenesis rather than osteogenesis. Meanwhile, osteoblasts, as well as osteocytes, showed increased apoptosis, decreased quantity, and multiple functional limitations including impaired mechanical sensing, intercellular modulation, and exosome secretion. Also, the bone resorption function of macrophage-lineage cells (including osteoclasts and preosteoclasts) was significantly enhanced, as well as impaired vascularization and innervation. In this study, we systematically reviewed the effect of aging on bone and the within microenvironment (including skeletal cells as well as their intracellular structure variations, vascular structures, innervation, marrow fat distribution, and lymphatic system) caused by aging, and mechanisms of osteoimmune regulation of the bone environment in the aging state, and the causal relationship with multiple musculoskeletal diseases in addition with their potential therapeutic strategy.

## 1. Introduction

Bone is a metabolically active connective tissue that provides muscle leverage to protect essential structures, provides structural support, promotes movement, stores minerals and growth factors, regulates mineral and acid-base homeostasis, and serves as a site for hematopoiesis [1]. Normal human skeletal development begins with bone formation and bone density increases with bone growth, peaking in late adolescence or early adulthood. Subsequently, bone loss occurs with age in both men and women, with accelerated bone loss in women during menopause[2]. With aging, bone quality and mineral content decrease, bone marrow fat level and bone turnover rate increase, and bone shape and structural composition change [3, 4]. Specifically, the substantial changes in the bone structure include decreased thickness and several bone trabeculae, loss of cortical bone, and increased porosity ratio [5]. Bone aging is often accompanied by osteoporosis (OP) [6]. As the support for connecting the two major systems of bone and bone marrow, the bone microenvironment mainly includes hematopoietic stem cells, SSCs, osteoblasts, bone lining cells, bone marrow adipocytes, bone macrophages, immune cells, and other cellular components, as well as the bone matrix, vascular structures, and other components [7].

According to the World Health Organization, the young people are under 44 years old, the middle-aged people are 45-59 years old, the young elderly are 60-74 years old, the elderly are 75-89 years old, and the longevity elderly are over 90 years old. World Population Prospects notes that approximately 1 in 11 people worldwide were 65 years of age or older in 2019. By 2050, one in six people worldwide will be over the age of 65 [8]. The global aging population has increased the incidence of osteoporosis and related fragility fractures, which seriously affects the quality of life and medical costs of patients [9]. With the increase of age, the bone homeostasis maintained by the complex balance between bone formation and bone resorption is dysregulated, leading to decrease in bone strength [10], and increase in the incidence of bone-related diseases.

We electronically searched PubMed using the keywords "bone", "osteocyte", "bone microenvironment", "autoimmunity", "osteoporosis" and "aging" up to November 2023 to collect studies on the effect of aging on bone. Additionally, the references of the included studies were assessed to supplement the acquisition of relevant literature. A total of 431 English-related articles were included.

In this review, we systematically reviewed the literature on the changes in bone cell structure and bone microenvironment (bone cells, vascular structures, bone marrow lymphatic vessels, and bone marrow fat) caused by aging, and their relationship with skeletal system-related diseases. In addition, we discussed mechanisms of osteoimmune regulation of the bone environment in the aging state and potential treatment options for osteoporosis caused by aging.

## 2. Age-related changes in bone cells

### 2.1 Bone marrow mesenchymal stem cells

Bone marrow mesenchymal stem cells (BMSCs) are a type of multi-potent cells, which play a central role in tissue regeneration, wound healing, and maintenance of tissue homeostasis [11-13]. They are involved in immune regulation, hematopoiesis, and bone formation [14]. BMSCs contain at least two subsets of bone stem cells and stromal cells. Skeletal stem cells differentiate into bone, cartilage, and fat. Stromal cells regulate immune function and inflammation, participate in wound healing, and promote angiogenesis [15]. Single-cell RNA sequencing (scRNA-seq) allows for transcriptome-wide analyses of individual cells, revealing exciting biological and medical insights [16]. ScRNA-seq analysis showed that the central region of BMSCs was marked by C-X-C motif ligand 12 (CXCL12) and leptin receptor (LepR). These BMSCs include multi-potent progenitor cells, pre-osteogenic BMSCs, and pre-adipogenic BMSCs (adipo-CAR cells and other adipogenic reticular stromal cells, known as MALPs) [17].

Similar to other types of stem cells, BMSCs are affected by aging. The expression of receptor activator of nuclear factor-κB ligand (RANKL) in senescent BMSCs, macrophage colony-stimulating factor (M-CSF) in osteoclast precursor cells [18, 19], and peroxisome-activated receptor γ (PPARγ) [20] were increased. The expression of forkhead box protein 1 (FOXP1) [21], discoid domain receptor 2 (DDR2) [22], osteoprotegerin (OPG) [18, 19], osteoblast-specific transcription factor Runt-related transcription factor 2 (Runx2), distal-less homeobox 5 (Dlx5), the osteoblast marker collagen [20], peroxisome proliferator-activated receptor-γ coactivator-1α (PGC-1α) [23] were reduced. Dlx-5 plays an essential role in promoting phenotypic expression of mature osteocytes [24]. Moreover, the glucose uptake, lactate secretion, adenosine triphosphate production and relative extracellular acidification rate of BMSCs in aged rats were decreased [25]. In addition, malondialdehyde levels were significantly increased, and total glutathione peroxidase, total antioxidant capacity, and superoxide dismutase activity were significantly decreased in aged BMSCs [23].

**Figure 1.**
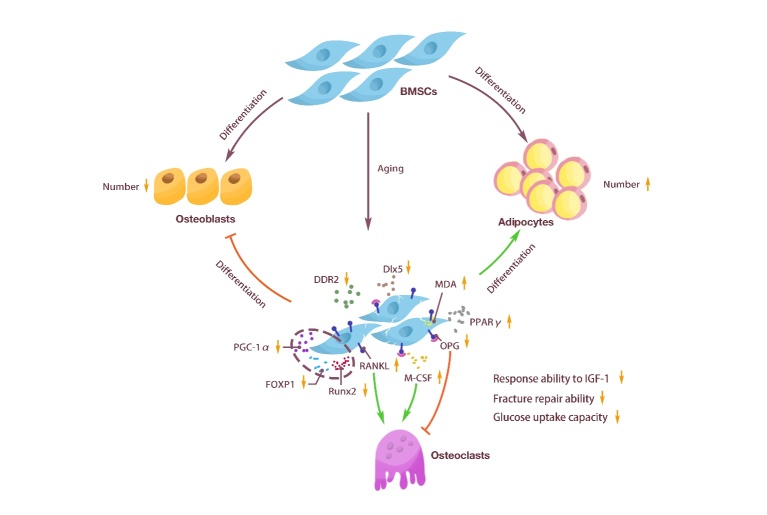
**Changes in BMSCs caused by aging**. BMSCs had the ability to differentiate into osteoblasts and adipocytes. With aging, the number of BMSCs decreased, the adipogenic differentiation enhanced, and the osteogenic differentiation weakened. The secretion of BMSCs was also regulated, such as decreased Dlx5, DDR2, PGC-1α, FOXP1, and Runx2, which inhibited the generation of osteoblasts. The up-regulation of PPARγ promoted the generation of adipocytes, the downregulation of OPG and the increase of M-CSF and RANKL promoted the generation of osteoclasts, and the up-regulation of MDA accelerated the aging of BMSCs. In addition, BMSCs cells showed a decreased ability to respond to IGF-1, fracture repair, and glucose uptake.

Aged BMSCs tend to differentiate into adipocytes [26]. Accumulation of adipose tissue and adipocytes has been observed in the bone marrow of aged people [27] and aged mice [28]. Aging may cause a decrease in the number of SSCs, and SSCs from elderly donors show a shorter maximum lifespan and a decrease in the ability to passage compared with young donors [29]. Yang *et al.* [30] studied the changes in phenotype and differentiation ability of SSCs derived from the bone marrow of aging population *in vitro* and found that aging affects the proliferation rate and osteogenic differentiation potential of SSCs. Zhong *et al*. [31] analyzed young adult and aged LepR-cre mice by scRNA-seq, and found that LepR-cre labeled most of the BMSCs and osteogenic lineage cells in adult long bones, and these BMSCs included adipogenic populations, such as adipo-CAR cells or MALPs. CXCL12+ LepR+ BMSCs give rise to osteogenic and adipogenic lineage cells, but the differentiation of these two lineages is mutually exclusive [32]. Deletion of LepR in BMSCs using Prrx1-cre promoted osteogenesis and decreased adipogenesis [33], whereas deletion of CXCL12 using Prrx1-cre and Osterix (OSX)-cre reduced osteogenesis and increased adipogenesis [34]. Mesenchymal progenitors lacking CXCL12 tend to form bone marrow adipocytes [35]. Aging induces CXCL12+ LepR+ BMSCs to differentiate into adipocytes rather than osteoblasts [28, 32], which could explain the enhanced bone marrow adipose tissue (BMAT) accumulation during aging [36]. In addition, BMSCs lose their responsiveness to insulin-like growth factor 1 (IGF-1) with aging [37], IGF-1 and its receptor can promote the proliferation and differentiation of adipose progenitor cells [38] and bone formation [39, 40]. Compared with young BMSCs, the fracture repair ability and osteoblast differentiation potential of aged BMSCs are significantly limited [41]. In old age, BMSCs lose their function and regenerative ability and undergo replicative senescence, which aggravates the progression of inflammation and cancer [42] (Fig. 1).

### 2.2 Osteoblast

Osteoblasts are spindle-shaped or cuboidal cells on the surface of bone [43], with a diameter of 10-15 micrometers [44]. They are so named because they regulate and affect the process of bone formation and reconstruction. They are mainly derived from SSCs in the inner and outer periosteum, and the matrix of the bone marrow [12]. Their formation requires differentiation of progenitor cells into proliferating pre-osteoblasts, osteoblasts producing bone matrix, and finally into osteocytes or bone-lining cells [45]. Active mature osteoblasts have large nuclei, enlarged Golgi structures and extensive endoplasmic reticulum, and they secrete type I collagen and other matrix proteins onto the bone-forming surface [46]. Specifically, osteoblasts first acquire a polarized phenotype, thereby enabling them to secrete bone matrix in a directed manner. With further differentiation, osteoblasts secrete calcium and phosphate ions to initiate the calcification process, thereby maintaining the integrity of the bone structure [47]. In addition, osteoblasts can regulate osteoclast function and maintain bone homeostasis through direct cell-cell contact, cytokine and extracellular matrix interactions [48].

The proliferation and activity of osteoblasts are regulated by various factors, among which Runx2 and OSX are two key transcription factors that promote the osteogenic differentiation of SSCs [49] and play an important role in osteogenic differentiation. Several other transcription factors are also important during differentiation. For example, interleukin-10 (IL-10), IL-11, IL-18 and interferon-γ (IFN-γ) can promote the generation of osteoblasts. However, anti-osteoblastic factors, such as tumor necrosis factor-α (TNF-α), TNF-β, IL-1α, IL-7, IFN-α, IFN-β, etc., down-regulate the production of osteoblasts [50]. In addition, some key signaling pathways, including Wnt, Notch, and bone morphogenetic protein (BMP), also play a regulatory role [51-53].

With aging, osteoblasts show increased apoptosis and decreased number [54]. Aging may cause a decrease in the number of SSCs. SSCs, as adipocytes and osteoblasts, have a common cell source. During the aging process, SSCs are more likely to differentiate into adipocytes [55]. Meanwhile, scRNA-seq analysis showed that CXCL12-creER+ BMSCs were transformed into osteogenic precursor cells in a process mediated by canonical Wnt signaling. Similarly, Dlx5-creER+ osteogenic progenitor cells were mediated by Wnt signaling but not by Sox9-related or RunX2-related pathways [56]. Wnt signaling pathway can inhibit adipogenesis and facilitate the differentiation of SSCs into osteoblasts [57], and has been confirmed to be generally down-regulated during aging [58], which leads to fat accumulation in the bone marrow cavity, thereby threatening the survival of osteoblasts [59].

**Figure 2.**
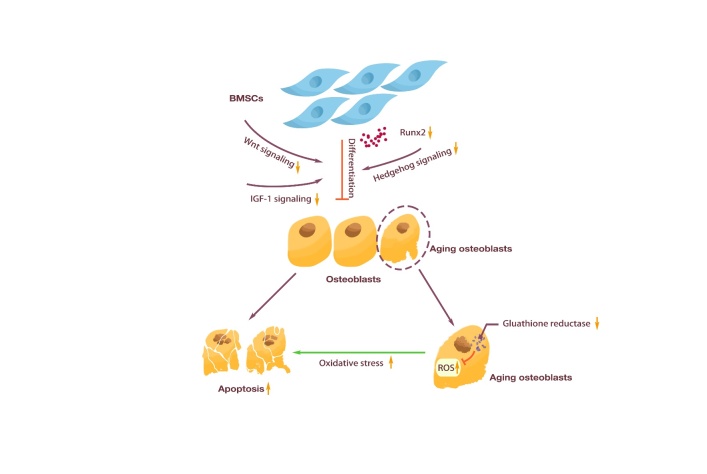
**Changes in osteoblasts caused by aging**. Osteoblasts were differentiated from BMSCs. With aging, the IGF-1 signaling pathway, Wnt signaling pathway, Hedgehog signaling pathway, and the ability of BMSCs to secrete Runx2 were weakened, which eventually led to the weakening of osteogenesis of BMSCs. In addition, decreased glutathione reductase in senescent osteoblasts led to ROS accumulation, which increased oxidative stress and ultimately accelerated osteoblasts apoptosis.

Aging can directly affect the differentiation and function of osteoblasts. Compared with osteoblasts cultured from young mice, osteoblasts from aged mice lack adaptive or metabolic flexibility to utilize exogenous substrates. The expression of oxidative stress genes was upregulated, and the expression of osteoblast-related genes was downregulated [60]. First, aging leads to a decrease in the lifespan of osteoblasts. Aging causes increased levels of oxidative stress in various organs, including bone [61]. This oxidative stress is a key mechanism of age-related bone loss in aged mice. Aged mice show increased osteoblast apoptosis and decreased bone formation, which are synchronized with elevated levels of reactive oxygen species, decreased glutathione reductase activity and increased phosphorylation of p53 and p66 [62]. Moreover, aging leads to decreased differentiation of osteoblasts. Roholl *et al*. [63] proposed that the number of osteoblasts decreases by more than ten times with aging, but the proliferation of osteoblast precursor cells is not affected by age. The loss of trabecular bone in aged rats is accompanied by a sharp increase in the ratio of osteoblast precursor cells to osteoblasts, indicating that weakened osteoblast differentiation during aging is a potential mechanism for impaired bone formation. Furthermore, aging leads to the reduction of growth factors such as IGF-1 and inhibits the IGF-1 signaling pathway, thereby affecting the function of osteoblasts. IGF plays an important role in promoting the differentiation of osteoblasts [64, 65], while aging can induce a decrease in the circulating level of IGF-1 [66]. Cao *et al*. [37] found that aging reduces the level of IGF-1 in aged mice and causes resistance of osteoblasts to IGF-1. The pro-proliferative and anti-apoptotic effects of IGF-1 were blunted in cells from aged mice. Osteoblasts derived from the elderly also show resistance to IGF [67]. In addition, Wnt signaling significantly weakens with age, thereby impairing osteogenic differentiation [68]. Similarly, Hedgehog signaling, which is responsible for regulating the balance of osteoblast/adipocyte differentiation, declines with age [69]. Hedgehog signaling inhibits MSCs differentiation to adipocytes, and it promotes their differentiation to chondrocytes and osteoblasts ^[70]^. Therefore, aging can affect the proliferation and function of osteoblasts by affecting the sources of differentiation, the activity of osteoblasts, and related signaling pathways and growth factors (Fig. 2).

### 2.3 Osteocyte

Osteocytes are the most abundant cell type in bone, which are distributed in the mineralized bone matrix, forming an interconnected network [71] and are ideal structures for sensing mechanical loads and controlling mineral homeostasis [72]. Osteocytes can sense load in various ways, such as through cell bodies, dendritic processes, and ciliary bending [73]. In addition, osteocyte networks can also detect microdamage and trigger its repair [74, 75].

In addition to coordinating the mechanical adaptation of bone structures, osteocytes coordinate the activity of osteoclasts [76] and are inducers of osteoclast activation [72]. Osteocytes are the main source of RANKL in osteoclast production, which acts through the RANKL/OPG mechanism [76]. Osteocyte-derived RANKL is required for age-related cortical bone loss [77], and mice lacking RANKL in osteocytes have increased BMD and reduced bone remodeling [78, 79]. Osteocytes can also regulate osteoblast activity by secreting stimulating factors, such as signaling lipids (e.g., PGE2) [80], growth factors (e.g., IGF-1) [81], glycoproteins (e.g., Wnts) [82], free radicals (e.g., NO) [83], and nucleotides (e.g., ATP) [84], thereby affecting osteoblast generation. One of the most potent signals that osteocytes produce to control osteoblast biology is a secretory inhibitor of Wnt signaling. Sclerostin and Dickkopf-1 (Dkk1) are strong antagonists of WnT-mediated osteoblast activity, and Dkk1 is also highly enriched in osteocyte populations [76]. In addition, osteocytes can remove and replace their peri-luminal and peri-tubular matrix [76, 85]. Osteocytes are the source of various products with the ability to regulate bone remodeling, including small-molecule mediators such as prostaglandins, nitric oxide, and nucleotides, as well as a wide range of cytokines and growth factors, such as IGF-1, vascular endothelial cell growth factor (VEGF), and transforming growth factor β (TGF-β) [86-91]. Meanwhile, osteocytes are also the main source of fibroblast growth factor 23 (FGF23) [71]. The number and function of osteocytes are altered with aging. Bone cell density is 30-40% lower in the elderly than in young people in their 20s and 30s [92, 93]. During aging, the accumulation of microdamage and the decrease in osteocyte density lead to a decrease in osteocyte space [94]. Compared with 5-month-old C57BL/6 mice, the number and cell density of osteocyte dendrites were significantly reduced in 22-month-old C57BL/6 mice [95]. This may be related to Sirt3, because Sirt3 depletion in osteocytes impairs osteocyte dendritic process formation and inhibits bone gain in response to exercise in vivo [96]. The number of osteocytes in telomere-induced foci (TIF) increased 6-fold with age. Using a threshold of ≥4 senescence-associated distension of satellites (SADS) per cell to define cellular senescence, the number of senescent osteocytes in the bone cortex of aged mice was found to be significantly increased [97]. In addition, the chromatin organization of osteocyte subsets in the bone cortex of aged mice was altered, with satellite heterochromatin around centromeres or extensive disintegration with SADS [98]. SADS are markers of aging regulator SMURF2 expression, oxidative stress, or oncogenic RAS-induced senescence [98]. In addition to changes in numbers, senescent osteocytes have other features including, but not limited to, impaired mechanical sensitivity [99], accumulation of cellular senescence [100], dysfunction of peri-lacunar/tubular remodeling [101], and degeneration of the lacunar-tubule network [102]. Moreover, some studies have shown that the mechanical responsiveness of bone is impaired [95], the number of osteocyte dendrites is negatively correlated with age, and the integrity of the osteocyte network is also damaged with age [103-105].

The RNA and protein expression levels of senescent osteocytes were also altered compared to young osteocytes. Among them, p16INK4a is one of the most well-studied markers of senescence and aging [106]. The expression of p16Ink4a in mouse osteocytes increased significantly after about 18 months of age, coinciding with the acceleration of bone loss [107]. The expression of p16Ink4a mRNA was significantly increased about 5-10 times with the aging of bone marrow cells, B cells, T cells, osteoblast progenitors, osteoblasts and osteocytes. The results were similar in female and male mice. Moreover, p21Cip1 mRNA levels in osteocytes of male mice also increased significantly with age [97]. P21 is another loop-independent kinase inhibitor [108] and canonical marker of senescence [109]. In addition, sost [110] p21 and p53, and several senescence-associated secretory phenotype (SASP) markers [97] were highly expressed in osteocytes of aged mice. Senescent cells secrete a large number of factors, including proinflammatory cytokines and chemokines, growth factors, angiogenic factors, and matrix metalloproteinases, which are collectively referred to as SASPs [111]. The levels of Atg7, Map1lc3a [97] and connexin43 (CX43) [112] were all significantly lower than those in young mice. Sost is a negative regulator of bone formation [110]. P53 triggers the apoptosis of genomic-damaged cells and induces cell cycle arrest. Atg7 [113] and Map1lc3a [114] are autophagy-related proteins. CX43 can preserve osteocyte viability and maintain bone formation to ameliorate age-induced cortical bone changes, thereby improving bone strength [112]. Aging leads to the accumulation of senescent cells in cortical bone, which increases the expression of Tnfsf11 encoding RANKL. When Tnfsf11 is absent in osteocytes, mice exhibit a severe osteoporotic phenotype due to the lack of osteoclasts [77].

**Figure 3.**
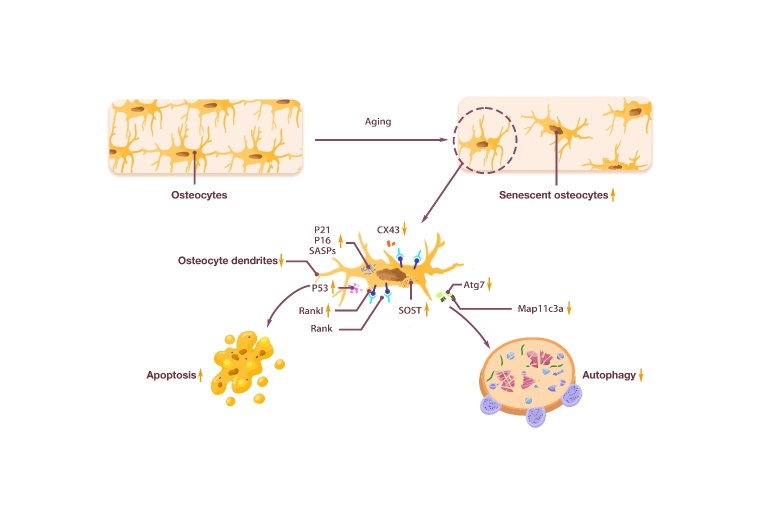
**Changes in osteocyte caused by aging**. Osteocytes had the function of sensing mechanical load and secretion. With aging, osteocytes number decreased, and senescent osteocytes showed reduced dendrites. In addition, the secretion of senescent osteocytes changed. The increase of P21, P16, and SASPs promoted osteocyte senescence, the increase of Rankl promoted osteoclast formation, the increase of SOST and the decrease of CX43 inhibited bone formation, the increase of P53 promoted osteocyte apoptosis, and the decrease of Atg7 and Map11c3a inhibited autophagy.

The above studies indicate that aging leads to reduced osteocyte number, impaired mechanical load sensing function and osteocyte network, as well as changes in RNA and protein expression in osteocytes (Fig. 3).

### 2.4 Osteoclast

Osteoclasts are hematopoietic cells derived from granulocyte-macrophage colony-forming unit [115], which diversify from the monocyte-macrophage lineage at the early stage of differentiation. Osteoclast formation and activity are controlled by various regulatory factors [116]. In the bone marrow, hematopoietic stem cells (HSCs) self-renew and differentiate into various hematopoietic cell types. It is not directly in the process of mature cells but in a hierarchical evolutionary tree divided into several types of spectrum limit progenitor cells, including multi-potent progenitors, common myeloid progenitors, etc., and osteoclast precursor cells [117]. Osteoclast precursor cells are further differentiated into mature osteoclasts under the combined action of M-CSF and receptor activators of nuclear factor-κb ligand RANKL and OPG [118]. M-CSF induces osteoclast precursor cells to respond to RANKL, and promotes osteoclast proliferation and survival [119]. However, RANK/RANKL signaling activates various downstream signaling pathways required for osteoclast development and fine-tunes bone homeostasis through crosstalk with other signaling pathways [120]. RANKL induces osteoclast formation when it binds to its receptor RANK in osteoclast precursors. Meanwhile, OPG binds to RANKL and prevents RANKL/RANK interaction, thereby inhibiting excessive osteoclast formation [121]. In addition, some proteins [122, 123], proinflammatory cytokines [124] and secreted products of osteoclasts [125] also directly or indirectly participate in the differentiation of osteoclasts.

Mature osteoclasts have special morphology and structure. As multi-nucleated giant cells, mature osteoclasts can have up to 59 nuclei [126]. During bone resorption, the osteoclast cytoskeleton reorganizes and polarizes the cellular resorption machinery to the osteocyte interface, forming an isolated resorption microenvironment [127] called the sealed or transparent zone. Simultaneously, a wrinkled border is formed at the edge of the sealing zone to secrete acid and collagenolytic enzymes [128, 129]. Osteoclasts dissolve calcium phosphate in bone by secreting proteins related to the acidification of the absorption gap and absorbing them into osteoclasts [130]. Meanwhile, type I collagen is degraded through the secretion of enzymes related to the degradation of bone extracellular matrix to promote bone dissolution [131].

**Figure 4.**
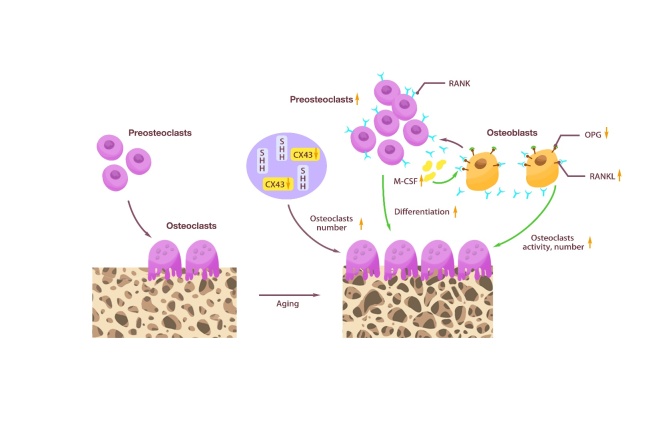
**Changes in osteocyte caused by aging**. Osteoclasts were differentiated from osteoclast preosteoclasts. With aging, the number of preosteoclasts increased and the differentiation into osteoclasts was enhanced. Osteoblasts secreted less OPG and more RANKL, promoting an increase in osteoclast activity and number. The increase of M-CSF induced preosteoclasts to respond to RANKL and promotes osteoclast proliferation and survival. CX43 reduction and SHH promoted an increase in osteoclast number.

Aging affects the proliferation and activity of osteoclasts. The number of osteoclasts in the diaphysis was significantly increased in aged mice, indicating excessive bone resorption [132]. Senescent cell-conditioned medium impairs osteoblast mineralization and enhances osteoclast progenitor cell survival, leading to increased osteoclast formation [133]. Ambrosi *et al*. [134] found that colony-stimulating factor 1 was significantly increased in 24-month-old mice, possibly leading to increased osteoclast formation. Cao *et al*. [135] showed that age-related bone loss in male C57BL/6 mice was related to the expression of RANKL and OPG. Compared with young mice, the expression of RANKL was higher and OPG was lower in aged mice, which increased osteoclast activity. Piemontese *et al*. [136] showed that osteocyte-rich bone preparations in aged mice increased RANKL transcripts, increasing the number of osteoclasts, leading to an imbalance in bone homeostasis. These results are consistent with in vivo osteocyte studies in the elderly, in which constitutive expression of RANKL increases with age, and OPG shows an age-related decline [18]. In addition, Kim *et al.* ^[77]^ found that osteoclasts did not increase with age in cortical bone of osteocyte (Dmp1-Cre) conditional knockout Tnfsf11 mice. Moreover, changes in some proteins during aging also affect the number and activity of osteoclasts. Davis *et al.* [137] found that aging causes a decrease in CX43 expression in bone, leading to an increase in the number and activity of osteoclasts on the surface of cortical bone in mice. Matsumoto *et al*. [138] found that Sonic hedgehog (SHH) protein was activated in osteoblasts at the site of dynamic fracture remodeling in young mice. At the same time, it directly stimulated the formation of osteoclasts in aged mice, resulting in imbalanced bone remodeling during aging. Therefore, aging leads to an increase in the number of osteoclasts by affecting the survival rate of osteoclast precursors, altering the differentiation bias of HSCs, and regulating factors related to osteoclast numbers (Fig. 4) (Table 1).

**Table 1:** Age-related changes in bone cells.

Cell types	Age-related changes
**BMSCs**	The number of BMSCs decreased, the adipogenic differentiation enhanced, and the osteogenic differentiation weakened. In addition, BMSCs showed a decreased ability to respond to IGF-1, fracture repair, and glucose uptake.
**Osteoblasts**	Osteoblasts showed increased apoptosis and decreased proliferation and function.
**Osteocytes**	Osteocytes number decreased, and senescent osteocytes showed reduced dendrites. In addition, the secretion of senescent osteocytes changed.
**Osteoclasts**	The number and activity of osteoclasts were increased, leading to enhanced bone resorption.

## 3. Age-related changes in cellular structure of bone cells

The changes in bone cells caused by aging are mainly reflected in the cell membrane, cytoplasm, nucleus, mitochondria, lysosomes, and exosomes. In the next paragraph, we described each of them separately.

### 3.1 Age-related changes in cell membrane components

Cell membranes are essential components of living organisms. They act as a highly selective permeability barrier, conferring internal characteristics on cells and organelles such as mitochondria, chloroplasts, and lysosomes, and have a central role in biological communication [139]. The composition of cell membranes changes with aging. The main molecular components of cell membranes consist of several structures. first, polar lipids, including phospholipids, glycolipids, and cholesterol, are organized into continuous bilayer layers that are liquid at immediate ambient temperatures; second, proteins (many of which are glycoproteins) interact with the lipid bilayer in different ways. Finally, nonpolar lipids and nucleic acids act as secondary components of the cell membrane [139, 140]. Phosphatidylcholine (PC) and phosphatidyl ethanolamine (PE) are glycerol phospholipids essential components of the cell membrane lipid bilayer. As the most abundant glycoprotein in the eukaryotic membrane, PC is considered an essential membrane lipid in lipoprotein assembly and secretion [141]. Studies have found significantly upregulated PC and downregulated PE contents in subjects with osteopenia and OP [142]. PE, another membrane aminophospholipid, is the second most abundant in eukaryotic cells and is a crucial participant in biological processes such as cell cycle regulation, autophagy, and regulation of membrane structure and properties [143, 144]. Previous research has shown that the cell content of glycerophospholipids, especially PE, is related to osteoclast generation and increases during osteoclast differentiation [145]. Palmitic acid (PA), a saturated fatty acid, is the activator of RANKL, which can induce the formation and differentiation of osteoclasts, even in the absence of RANKL. A previous study found that PA can reduce the function of osteoblasts in vitro and bone formation markers in vivo in a dose-dependent manner [146]. Moreover, in senescent cells, most phospholipids, including phosphatidylcholine, phosphatidylglycolamine, phosphatidylglycerol, and sphingolipids, are significantly increased, while phosphatidyl acid, phosphatidylinositol, and phospha-tidylserine are decreased [147]. Also, the ratio of phosphatidylinositol to phosphatidylserine increases in late-passage samples. In addition, during the passage of human cells, phosphatidylcholine and phospha-tidylethanolamine containing essential polyunsaturated fatty acids 20:4n-6 increase, while substances containing monounsaturated fatty acids decrease [148].

As a critical and typical cell membrane, the nuclear membrane significantly changes during aging. The nuclear membrane is the protective barrier of the eukaryotic cell genome and the communication interface between the nucleus and cytoplasm, which is mainly composed of three parts: a nuclear layer, a double membrane, and the nuclear pore complex [149]. The double membrane consists of the inner and outer nuclear membrane, separated by the perinuclear space [149], which acts as a unit to maintain the dynamic connection between the cytoskeleton and chromatin [150-152]. A-type lamins are an essential component of the nuclear membrane that participate and in maintaining the shape and strength of the nucleus, as well as in many nuclear processes, including DNA replication and transcription [153]. Nuclear skeleton laminin A/C provides mechanical elasticity to the nucleus [154] and regulates SSCs differentiation and skeletal phenotypes. Its expression change is related to age-related bone changes [155]. A comparison of laminin A/C expression in osteoblasts of young and old C57B/L6 mice showed that the expression level of laminin A/C in old mice was significantly reduced [156]. In addition, a mutant form of laminin A/C was observed on the senescent nucleus of a skin fibroblast cell line from an elderly individual [157], and the mutation in this gene has been associated with Hutchinson-Gilford syndrome [158]. During aging, laminin A/C affects the ability of SSCs to differentiate into osteoblasts, promotes lipogenesis, and affects the function and survival of bone cells [155], such as the mechanical conduction function of bone cells [159].

### 3.2 Age-related changes in the cell cytoplasm

The cytoplasm is a gel-like crowded intracellular environment composed of various macromolecules, organelles, cytoskeletal networks, and cytoplasmic fluid [160]. Therefore, decreased cytoplasmic ribosomal biogenesis and accumulation of rRNA precursors during aging, especially ribosomal protein L29 *in vitro* and *in vivo*, can be used as accurate biomarkers of aging cells [161].

MiRNAs, as substances in the cytoplasm, have been proven to be related to cell aging and tissue function decline in an increasing number of studies. Increased expression of miR-195 has been found in BMSCs of aged mice and telomeres, which are essential for maintaining telomerase reverse transcriptase, and have been identified as targets of miR-195 [162]. Contrarily, the expression levels of miR-543 and miR-590-3p reduced in aged human skeletal stem/stromal cells (hSSCs), and their overexpression could reverse the aging phenotype of SSCs [163]. In addition, during aging, the difference occurs in RNA of human bone marrow mesenchymal stem cells (hBMSCs) compared to primary generation cells. For example, the expression profiles of miRNA, snRNA, snoRNA, piRNA, and rasiRNA in the 10^th^ generation of hBMSCs were shown to be significantly different from those in the 1^st^ generation. In the 10th generation of hBMSCs, 23 miRNAs were upregulated, and one was downregulated [164]; among those, MiR-204 showed the greatest upregulation [165] and could inhibit osteogenic differentiation of BMSCs by targeting Runx2 [166]. Moreover, a study found that miR-183 increases with aging [164]. Aquino-Martinez *et al.* [167] reported that miR-219a-5p is decreased in the bones of 24-month-old mice compared with 6-month-old young mice, while the expression of Rorβ is increased. Furthermore, Rorβ functionally inhibits bone differentiation and Runx2- and β-catenin-dependent transcriptional activation at the molecular level. In addition, inhibition of miR-219a-5p in mouse cranial osteoblasts leads to increased expression of Rorβ. Global deletion of Rorβ in mice leads to improvements in bone mass, microstructure, and strength and is associated with increased bone formation, decreased resorption, and increased Wnt pathway activation [168-170]. More importantly, the expression of miR-219a-5p was also reported to be reduced in bone biopsies of older adults compared with those of the younger group [167].

NAD+-dependent deacetylases Sirtuin-3 (Sirt3), found in exist in the cytoplasm of bone marrow cells, are associated with aging and related disease processes [171]. As aging occurs, Sirt3 expression increases in bone marrow cells, which suggests that Sirt3 promotes age-related adipogenesis and osteoclasts [172]. DDR2 was observed in aging hBMSCs [22], just as fibroblast growth factor-2 was observed in aging hSSCs [173], FOXP1 in aging mouse BMSCs [21], a cluster of differentiation CD34 and CD19/CD14 in aging hBMSCs [23], OPG [18, 19] in aging human bone marrow cells, and decreased expression of 2-oxoglutarate and Fe^2+^-dependent hydroxylase (Alkbh1) [174] in BMSCs of aging mice. However, there were increased expression levels of RANKL, M-CSF, CD73, CD90, and malondialdehyde in hBMSCs47, PC-PLC activity in BMSCs, and integrin β 4, caveolin-1, reactive oxygen species (ROS) [175], and senescence-associated β-galactosidase (SA-b-Gal) activity in aging human bone marrow cells [164]. Besides, FOXP1 depletion was also found to significantly accelerate the aging of mouse BMSCs *in vivo*, resulting in lower osteogenic potential of SSCs than adipogenesis potential. Alkbh1 regulates the fate of BMSCs and bone fat balance in bone aging, inhibits adipose differentiation of BMSCs, and promotes osteogenic differentiation [174]. Also, many iNOS/CD206 double-positive cells have been found in the nonsecretory type (M0) of bone marrow-derived macrophages in aged mice. In contrast, the loss of Arg1/CD206 mRNA expression has been found in the anti-inflammatory type (M2) of bone marrow-derived macrophages in aged mice. Compared with M0s of myelogenous macrophages of young mice, the expression of iNOS and CD206 in myelogenous macrophages of old mice was found to be increased, and most significantly, the number of M0s of myelogenous macrophages of old mice with double-positive iNOS and CD206 was increased in M0s of old mice [176]. Compared to early passage cells, the osteocalcin levels of osteoblasts at late senescence were found to be significantly reduced, indicating that the function of osteoblasts is related to age [177]. In addition, the expression of p21 in old mice was found to be significantly higher than that in young male mice, and the expression of p53 in bone cells and myelocytes was significantly higher than that in young female mice. In contrast, the levels of Atg7 and LC3 in old mice's bone cells and bone cells were significantly lower than those of young mice. Moreover, cultured primary osteocytes from aged mice expressed a higher level of telomere dysfunction-induced foci, cell cycle inhibitors p16Ink4a, p21, and p53, and a variety of SASPs [97]. Hamrick *et al.* [107] and Glatt *et al.* [178] demonstrated that the expression of p16Ink4a in mouse bone cells was significantly increased after approximately 18 months of age, which coincides with accelerated age-related bone loss in both female and male mice. This can maintain bone cell vitality and bone formation to improve age-induced cortical bone changes and increase the expression of connexin 43 in bone cells, decreasing bone strength with age [112]. In addition, osteoblasts of older mice overexpress sclerosclerin, a negative regulator of bone formation [110].

### 3.3 Age-related changes in the cell nucleus

The nucleus is the interphase form of chromosome localization and chromatin structure [179], which integrates cellular and environmental signals [180].

Senescent cells differ from resting and terminally differentiated cells in their unique phenotypic features, which manifest as profound chromatin and secretory changes caused by nuclear DNA damage and mitochondrial dysfunction [98, 181, 182]. From *Caenorhabditis elegans* to humans, heterochromatin is lost with age, including essential and common heterochromatin regions such as telomere ends and pericentromeric regions [183]. Furthermore, new heterochromatin regions, known as senescence-related heterochromatin spots, also appear in senescent cells [184, 185]. These regions are transcriptionally inactive and may contribute to the stagnation of the cell cycle during aging [186]. In addition, cellular senescence induces changes in nuclear morphology, from several dense nucleoli in proliferating cells to one enlarged nucleolus in senescent cells stalled in the G1/S phase of the cell cycle [161, 186, 187]. Studies have also found increased DNA damage markers in aged mice, such as increased levels of phosphorylation of the serine residue of H2AX, p53 phosphorylation, and p21 expression, as well as increased expression of the GATA4 gene and increased nuclear factor kappa B and activated SASP [188].

Telomeres are repetitive DNA sequences found at the ends of chromosomes protected by protein caps and maintained by telomerase. Telomere length shortens with cell division and increases with age [189]. Telomere shortening is associated with the replicative senescence phenotype of human osteoblasts and SSCs in vitro [190-192]. In mouse SSCs, telomere shortening restricts the differentiation of osteoblasts, induces osteoblastic senescence, and reduces cell proliferation [193]. In addition, shortened telomeres lead to the activation of a DNA damage repair system that recognizes telomere ends as double-strand breaks [194]. Unrepaired DNA damage enhances the process of aging, which is also dependent on the p53 and p21 pathways [194-196].

### 3.4 Age-related changes in cell mitochondria

Mitochondria are the central hubs of cell metabolism and signal transduction, regulating metabolism and cell protein deposition, and their activities are closely related to senescence [197]. With increasing age, DNA mutations in the mitochondria gradually increase [198, 199], and alterations related to morphology and function, including reduced biogenesis, mitochondrial dysfunction, and biological energy failure, accumulate [200]. This mitochondrial damage is attributed to various mechanisms, such as increased mitochondrial DNA damage and accumulation of ROS production, decreased respiratory chain protein levels, deficiency in mitochondrial phagocytosis, dysfunctional mitochondrial unfolded protein response, and decreased mitochondrial contact in the endoplasmic reticulum [197, 201-203]. These defects drive aging through mitochondrial dysfunction, the interaction between ROS and DDR, as well as abnormal signaling through telomere shortening or replicating aging pathways, ultimately disrupting bone cell function, especially stem cell function [200].

The impact of aging on mitochondrial function involves changes in mitochondrial respiration, ATP reduction, and metabolites[204]. This change is reflected in the increase in glycolysis during the aging process. Compared with 13-month-old mice, the bone tissue of 18-month-old mice shows higher glycolytic metabolism, accompanied by a decrease in the level of metabolites in the tricarboxylic acid cycle [205]. Mitochondria also produce ROS when they generate ATP through oxidative phosphorylation [204, 206], and the level of ROS produced during an organism's lifetime is directly related to the rate of aging [207], which suggests a close relationship between senescence and mitochondria. In an introductory genetic study of bone mineral density in aging C57BL/6 mice, it was found that oxidative stress increases, which is related to the increase in osteoblast apoptosis and the decrease in osteoblast number and bone formation rate, which, in turn, promotes bone senescence [208]. Mechanistically, during this process, ROS antagonized the skeletal effect of Wnt/β-catenin *in vitro* by transferring β-catenin from Tcf to FoxO-mediated transcription [62]. In addition, mitochondrial ROS can damage nuclear DNA, thus activating the DNA damage response that induces senescence [209, 210].

Defects in the mitochondrial mechanism observed during aging are essential for bone loss and vulnerability. The mitochondrial respiratory chain provides 95% of the energy needed for cell survival through the activity of a complex of five mitochondrial respiratory enzymes in the inner mitochondrial membrane. Disruption of mitochondrial respiratory chain transmission can disrupt mitochondrial homeostasis, which results in an adverse bone phenotype. This dysfunction is a major cause of bone aging and metabolic imbalance [211]. Mitochondrial energy metabolism in BMSCs maintains their viability, osteogenic ability, and lipid-forming potential [212]. Previous studies [213] found lower abundance and maturity of mitochondria in elderly BMSCs with defective differentiation, while changes in ultrastructure were not as evident as those in young BMSCs. Both stromal cells from aged mice and mature matrix-secreting osteoblasts exhibit mitochondrial dysfunction [60]. Moreover, oxidative metabolism in aging bone is impaired, leading to glycolytic transformation, nucleotide imbalance, and a decreased NAD+/NADH ratio. These factors converge on the mitochondria of mouse bone cells and disrupt the integrity and function of the mitochondrial membrane, opening a large but nonselective permeability transition pore regulated by cyclophilin D, destroying the integrity of the mitochondrial membrane and damaging its function, leading to bone loss [205].

### 3.5 Age-related changes in cell lysosomes

Lysosomes are monolayer organelles formed by 7-10 nm thick lipid membranes with an acidic intracavity Ph [214]. As the main "digestive center" in cells, lysosomes can effectively degrade biomolecular substances such as polysaccharides, nucleic acids, proteins, fats, pathogens, and aging organelles to maintain cell self-renewal and energy requirements [215]. Substances from different sources are transported to lysosomes for degradation in different ways. For example, extracellular substances, such as pathogens and related toxins, can be transported to lysosomes for degradation through endocytosis [215]. At the same time, unfolded or misfolded intracellular cytoplasmic macromolecules and whole organelles are captured and transported to lysosomes for degradation, mainly through autophagy [216].

Previous studies [217-219] have shown that the increase in the number and activity of lysosomes is associated with replicative aging. The activity of the lysosome is reflected in the activity of the enzymes in the lysosome. SA-β-gal is a lysozyme [220] widely used as a biomarker for senescent cells [221-223]. The optimal pH value of SA-β-gal in different cells differs, and the optimal pH value in young or immortal cells is 4 [224]. However, the optimal pH in senescent cells is 6 [223-225]. α-fucosidase, an age-related lysosomal enzyme, is a more sensitive marker of aging that can also be used as an age-related marker when SA-β-Gal expression is low [226]. In addition, tartrate-resistant acid phosphatase (TRAP) is another representative enzyme of lysosomes and is an iron-containing protein highly expressed in osteoclasts [227, 228], osteoblasts [229, 230], macrophages [231], and dendritic cells [232, 233]. Osteoclasts secrete this enzyme during bone resorption, and the activity of the TRAP enzyme in serum is enhanced under increased bone resorption [234, 235]. This enzyme has been associated with aginge, with a higher turnover rate at eight weeks mouse compared with six months mouse, possibly because the growth rate of bone decreases with age [236]. In addition, this enzyme's activity is higher in younger subjects aged 0-18 years old due to more active bone remodeling processes associated with growth [237]. Moreover, another study found that the bones of TRAP-deficient mice are fatter and shorter than those of normal mice, with a thicker cortex [238], altered metaphyseal trabeculae, abnormal shape, wider epiphyseal growth plates, no tissue, and delayed cartilage mineralization [239, 240].

### 3.6 Age-related changes in cell exosomes

Exosomes are small vesicles with a diameter of 40-100 nm that are derived from the membranes of poly vesicles and released by various cells, such as osteoblasts [241, 242]. They carry a variety of functional biomolecules, including proteins, mRNAs, microRNAs, and lipids, which play a crucial role in intercellular communication [243-246].

There is a difference in the release of exosomes between senescent cells and young cells. Senescent cells secrete more exosomes than young cells, especially in the case of cancer [247]. In the mouse bone microenvironment, exosomes regulate various cellular processes by paracrine signaling, including osteocyte differentiation and bone structure maintenance [248]. Multiple studies [248-250] have also reported that exosomes released by osteocytes in the bone microenvironment promote multiple cascades of intracellular or intercellular signaling mechanisms by targeting the same cells and adjacent cells or reaching distant organs through circulation.

**Figure 5.**
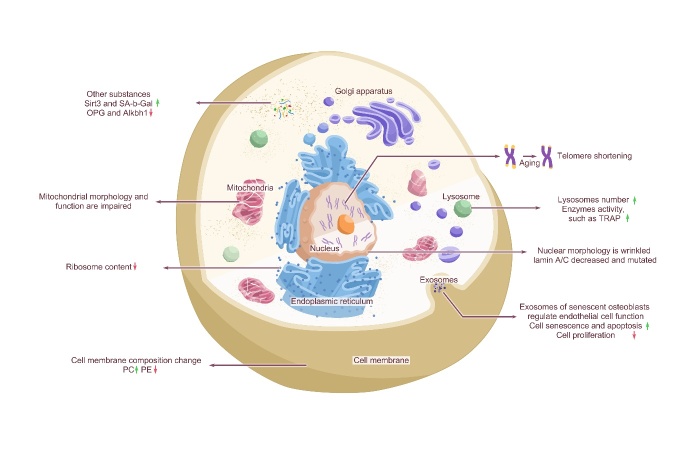
**Changes in bone cells caused by aging**. Cell membranes were essential components of living organisms. With aging, cell membrane components of bone cells changed, PC increased, PE decreased, and the lamin A/C component of the nuclear membrane decreased and mutated; in the cytoplasm, the increase of Sirt3 expression promoted adipogenesis and osteogenesis, and SA-b-Gal, a cellular senescence marker, was increased. The decrease of OPG was conducive to osteoclast formation. The decrease of Alkbh1 inhibited the osteogenic differentiation of BMSCs, but promoted their adipogenic differentiation. Moreover, nuclear morphology was shrunk, and telomere length was shortened. Also, mitochondrial morphological and functional defects were increased, including reduced biogenesis, mitochondrial dysfunction, and bioenergetic exhaustion, and the number of lysosomes and the activity of enzymes within lysosomes were increased, such as TRAP. Senescent osteoblast exosomes regulate endothelial cell function, promote cell senescence and apoptosis, and reduce cell proliferation.

Exosomes from different cells contain different components and have different functions. Several studies [251, 252] have demonstrated that long noncoding RNA metastasis-associated lung adenocarcinoma transcripts in mouse BMSC-derived exosomes can enhance bone repair by regulating osteoclast precursors and increasing osteoblast activity. Exosomes derived from MSCs can increase the migration of MSCs to the fracture site and enhance osteogenic differentiation and angiogenesis [9]. Zhang *et al.* [253] showed that exosomes derived from mouse BMSCs promote osteogenesis and angiogenesis through the BMP-2/Smad1/Runx2 signaling pathway, thereby improving fracture healing. Zhai *et al.* [254] found that hBMSC-derived exosomes could upregulate osteogenic miRNAs (Hsa-miR-146a-5p, Hsa-miR-503-5p, HsamiR-483-3p, and Hsa-miR-129-5p) or downregulate anti-osteogenic miRNAs (Hsa-miR-32-5p, Hsa-miR-133a-3p, and Hsa-miR-204-5p) to promote bone formation by activating the PI3K/Akt and MAPK signaling pathways. At the same time, Jia *et al.* [255] found that BMSC-derived exosomes in young mice could upregulate the expression of osteogenic genes, promote the proliferation of BMSCs, and accelerate the establishment of bone in elderly mice. The transfer of miR-214-3p from osteoclast-derived exosomes inhibits osteoblasts and bone formation [248]. Furthermore, Deng *et al.* [249] reported that the exosomes released from osteoblasts of the UAMS-32P cell line included the RANKL protein, and the RANK signal in the osteoclasts precursor was activated through receptor-ligand interaction (RANKL-RANK), leading to osteoclast formation. Senescent osteoblasts can regulate endothelial cell function, promote cell senescence and apoptosis, and reduce cell proliferation through the exosome pathway. MiR-139-5p was highly expressed in senescent osteoblasts and exosomes. After treatment with senescent osteoblast-derived exosomes, miR-139-5p was upregulated in endothelial cells [256]. At the same time, mouse osteoblasts secrete microbubbles carrying RANKL protein, which can promote the development of osteoclasts [249]. Moreover, the promotion of mouse osteoblast differentiation is modulated by exosomes secreted by osteoblasts and BMSCs [251, 257] (Fig. 5).

**Table 2:** Age-related changes in cellular structure of bone cells.

**Cellular structure of bone cells**	Age-related changes
**Cell membrane components**	The composition of the cell membrane was changed, the content of PC was significantly up-regulated, and the content of PE was significantly down-regulated. Moreover, in senescent cells, most phospholipids, including phosphatidylcholine, phosphatidylglycolamine, phosphatidylglycerol, and sphingolipids, are significantly increased, while phosphatidyl acid, phosphatidylinositol, and phosphatidylserine are decreased.
**Cell cytoplasm**	The contents of multiple miRNAs in the cytoplasm were changed, with increased expression of miR-195 and decreased expression of miR-543, miR-590-3p and miR-219a-5p. In addition, the expression of a variety of substances was changed. For example, the expression of Sirt3, RANKL, M-CSF, CD73, CD90, malondialdehyde, P21, P53, p16, and SASP increased, while the expression of DDR2 FOXP1, CD34, CD19, CD14, OPG, 2-ketoglutarate, and Alkbh1 decreased.
**Cell nucleus**	Chromosome telomeres were shortened, heterochromatin regions appeared, nuclear morphology changed to an enlarged nucleolus, and DNA damage markers increased.
**Mitochondria**	DNA mutations in mitochondria gradually increased, and morphological and functional changes were shown by disruption of mitochondrial membrane integrity, reduced biogenesis, mitochondrial dysfunction (including mitochondrial respiration, ATP reduction, and metabolite changes), and bioenergetic exhaustion.
**Cell lysosomes**	The activity of lysosome was changed, which was reflected in the activity of SA-β-gal and TRAP.
**Cell exosomes**	Both the number of exosomes secreted and the type of exosomes were changed in aging.

## 4. Age-related changes in the bone microenvironment

The changes in the bone microenvironment caused by aging involve the apoptosis and autophagy of bone vessels, bone fat, bone nerves, and cells. In the next paragraph, we described each of them separately (Table 3).

### 4.1 Age-related changes in the skeletal vascular system

The skeletal vascular system has a central role in maintaining the osteogenic and hematopoietic microenvironments, providing many inducers of vascular secretion signals, oxygen and nutrients [258]. Vessels in bones are divided into two main categories: H-type and L-type. H-type vessels, a recently discovered subtype of bone vessels, show high levels of endothelial mucin and CD31, which span the bone and form intracranial vessels. Osteoprogenitor cells selectively surround H-type endothelial cells, while L-type endothelial cells do not. H-type vessels are more abundant in young mice at four weeks than in adult mice at 11 weeks.

In contrast, 70-week-old mice have almost no H-type vessels [259]. Similarly, Lu *et al.* [260] found that the fracture vascular density and cartilage volume in old and middle-aged mice were significantly reduced compared with those in young mice. With the decrease in H-type vessel density, aging bones show a decrease in blood flow, which may lead to metabolic changes in the bone marrow microenvironment [258, 261].

Bone marrow endothelial cells are critical in osteogenesis, bone angiogenesis, and hematopoiesis. These cells are located at the interface between the vascular lumen and the bone marrow; they respond to various chemical or mechanical stimuli and regulate cell crosstalk between the two compartments [262]. Aging reduces the number of H-type vessels, decreases bone progenitor cells, osteoblasts, and BMD, and decreases endothelial expression of hypoxia-inducible factor-1α, resulting in the loss of H-type endothelium. The proliferation of H-type vascular subsets of endothelial cells is high in young mice. However, it decreases rapidly in adults, while there is no significant difference in the proliferation rate of L-type endothelial cells between young and elderly animals [259]. Age-related loss of H-type endothelial cells also seems to have an essential role in the pathogenesis of osteoporosis [263]. Bone marrow epithelial cells of older mice express significantly lower levels of hematopoietic factors, including SDF1 (CXCL12), SCF, and Notch ligand, which are critical for HSC homeostasis [259, 264]. Activation of endothelial Notch signals enhances blood flow into the bone and increases the abundance of HSCs [265]. The endothelium also shows significant morphological changes, including increased vascular dilation, leakage, and impaired vascular integrity [264].

### 4.2 Age-related changes in adipose tissue

Adipose tissue is a terminally differentiated organ providing energy and an endocrine organ with various endocrine, autocrine, and paracrine functions [266]. Bone marrow adipose tissue, as a unique fat pool located in bone, may contribute to local and systemic metabolic processes [267] and protect human bone osteoblasts from lipid toxicity [268].

Studies have found that fat content and distribution change with age. For example, multiple studies [27, 28, 269, 270] have demonstrated that bone marrow fat increases substantially with age. According to statistics, the total fat content of men and women doubles between 20 and 50 [271]. Fat is redistributed with age and subcutaneous and visceral fat decreases. At the same time, some organs are infiltrated by newly formed fat [272, 273] and incredibly long bones [274], and fat infiltrating bone marrow secretes a large number of fatty acids, which affects osteoblast differentiation, function, and survival [20, 275]. Moerman *et al.* [20] found that fatty acid binding protein aP2, a marker of the adipocyte phenotype, is highly expressed in BMSCs of aged mice. At the same time, the mass generation of adipocytes leads to the unbalanced differentiation of BMSCs, affecting osteoblast differentiation, function, and survival [13, 276]. Moreover, lipid droplets accumulate in early stromal cells and mature osteoblasts in aged mice, further indicating that lipid content is increased within the bone cortex in aged mice [60].

In addition, age-related bone mass loss occurs with the expansion of bone marrow fat [59, 277], which is associated with increased expression of miR-188 in the BMSCs of aging mice [278]. Lipogenic regulatory proteins also have an essential role in this process, including glycoproteins, synthesizer-3, and PPARγ [279]. The nuclear transcription factor PPARγis an effective mediator of adipogenesis associated with CCAAT enhancer binding protein α (C/EBP-a) [280]. In humans, Runx2 drives the differentiation of BMSCs into adipocytes and osteoblasts. There are two subtypes of PPARγ, PPARγ1 and PPARγ2. PPARγ1 is widely expressed throughout the body, while PPARγ2 is mainly expressed in adipose tissue [281]. The expression of PPARγ2 in BMSCs increases with age [279] and activates lipogenesis at the expense of reducing osteogenesis [282]. The transcriptional coactivator with PDZ-binding motif (TAZ), which activates Runx2, is a cytoplasmic 14-3-3 binding protein. It may regulate BMSC differentiation by inhibiting the transcription of PPARγ-dependent genes in favor of osteoblast generation [283-286]. Thus, when lipogenesis becomes common, TAZ may be downregulated by various conditions during aging.

**Table 3:** Age-related changes in the bone microenvironment.

Bone microenvironment	Age-related changes
**Skeletal vascular system**	The number of H-type vessels, the proliferation ability of vascular subsets, and the expression of hematopoietic factors were decreased. The endothelium also showed marked morphological changes, including increased vasodilation, leakage, and impaired vascular integrity.
**Adipose tissue**	Bone marrow fat increases and accumulates in the bones. Fat infiltrating bone marrow secreted large amounts of fatty acids, which affected the differentiation, function, and survival of osteoblasts.
**Skeletal nerve fibers**	Some functional innervation was diminished, but the density of sensory nerve fibers that transmitted noxious stimuli remains unchanged with age.
**Bone marrow lymphatic vessels**	The increase of P16 and P27 and the decrease of Ki67 and Vegfr3 resulted in the inhibition of lymphocyte proliferation.

Oxidative stress also has a vital role in promoting the production of bone fat. Oxidative stress has long been considered a significant cause of aging [287]. The bone marrow microenvironment, which is similar to many other organs and tissues, is exposed to oxidative stress during aging. Aging itself may be conducive to lipogenesis, combined with cellular resistance to oxidative stress and apoptosis, leading to fat accumulation in aging bone [288]. In their study, Almeida *et al.* [289] proved that older mice have elevated levels of oxidized lipids that act as free radicals, increase PPARγ expression, and promote apoptosis of osteoblast lineage cells compared to younger mice. In addition, heme oxygenase-1 (HO-1), a known reagent for neutralizing oxidative stress [290], has been found to affect hBMSCs differentiation, and inhibition of HO-1 significantly increases PPARγ expression and adipogenesis in hSSCs [291]. In short, increased intracellular oxidative stress may be one of the main drivers of the transition from osteogenic differentiation to lipogenesis during aging.

### 4.3 Age-related changes in skeletal nerve fibers

The peripheral nervous system involves bone metabolism, osteogenic differentiation of precursor cells, bone mineralization, and bone remodeling [292, 293]. Nerves innervate bone, yet different parts of the bone are innervated to different degrees. The higher the metabolic activity of the bone, the richer the sensory and sympathetic innervation [294]. Sensory and sympathetic nerve fiber networks are dense in periosteal and bone trabeculae in bones but less dense in cortical bone, bone marrow, and epiphyseal growth plates [295-299]. At the same time, a previous study [298] has also shown that sympathetic and sensory nerve fibers often innervate trabecular bone, the periosteum, and fracture calluses.

The nervous system is of essential importance for regulating bone growth and limb formation [300], and peripheral neurodegeneration may promote age-related bone loss [301]. Singh *et al.* [302] and Herskovits *et al.* [303] found that the sympathetic nerve regulates the activity of osteoblasts in mice, promotes bone resorption by increasing the expression of RANKL [304], and affects the formation of osteoclasts by regulating the expression of osteoclast differentiation factor/osteoprotectin ligand (ODF/OPGL) and osteoprotectin/osteoclastogenesis inhibitory factor (OPG/OCIF) produced by osteoblasts and stromal cells [304, 305]. When the sympathetic nerve is removed, it results in a loss of nutrition and affects osteogenesis [302, 303]. However, using β-blockers benefits bone mass and reduces fracture risk [306], which indicates that the sympathetic nerve has a complex and vital role in regulating bone formation.

Moreover, loss of articular sensory innervation during aging accelerates degenerative cartilage changes and promotes the development of spontaneous osteoarthritis in mice [307, 308]. In addition, capsaicin-sensitive sensory neurons have an active role in maintaining bone homeostasis, such as maintaining trabecular bone integrity and bone mass in the epiphysis of the tibia and femoral shaft. When these neurons are damaged, bone resorption is enhanced, new bone formation is reduced, and trabecular connection and thickness are lost, ultimately leading to increased bone fragility [309]. With the aging process, partial innervation weakens. Jimenez-Andrade *et al.* [310] showed that the density of sensory nerve fibers that transmit harmful stimuli remains unchanged with age. There was no significant difference in the density of calcitonin gene-related peptide (CGRP) and 200 kDa sensory nerve fibers innervating the femur in the femoral periosteum of 4-, 13-, and 36-month-old mice.

Studies have shown that sensory neurons can maintain bone homeostasis by producing various neuropeptides [299, 311, 312]. Among neuropeptides, CGRP has a vital role in bone growth, repair, and performance [313, 314]. CGRP directly promotes human osteoblast differentiation and inhibits osteoclast formation by interacting with functional CGRP receptors expressed by cells, possibly through the Wnt/β-catenin signaling pathway [313]. In addition, CGRP may also have an indirect role by enhancing the secretion of osteoblast cytokines in human endothelial cells and osteoblast precursor cells [314]. CGRP inhibits the number of osteoclasts and increases the expression of BMP-2 and other growth factors through the OPG/RANKL ratio, which affects the role of inferior alveolar nerve transection in bone [315]. Vasoactive intestinal peptide (VIP) regulates osteoclast formation [305] and activates the PKA/CREB pathway in mouse cranial osteoblasts, bone marrow, and stromal cells by increasing cAMP [316, 317]. In addition, it influences the expression of RANKL/OPG to inhibit bone resorption activity [318]. Neuropeptide substance P (SP) mainly regulates is mainly involved in regulating bone formation through concentration changes. When the concentration exceeds 8-10 M, it stimulates the differentiation of osteoblasts and bone matrix mineralization in mice [318, 319]. When SP is absent, adult mice with endochondral ossification show adverse effects on bone structure, reduced pain sensitivity, and mechanical stability of bone [320]. Also, SP promotes bone differentiation by activating the Wnt/β-catenin signaling pathway [321], and by activating this pathway, SP induces the proliferation of mouse BMSCs [322]. In addition, epinephrine and isoproterenol regulate human osteoclast generation [304].

### 4.4 Age-related changes in bone marrow lymphatic vessels

**Figure 6.**
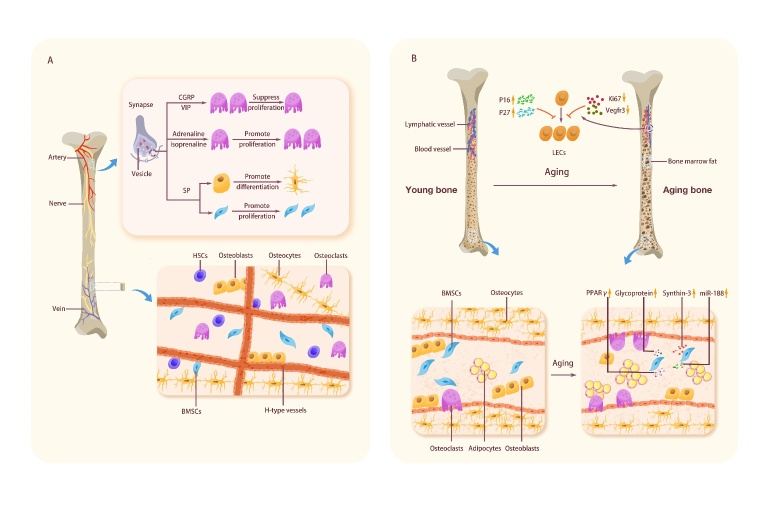
**Changes in bone nerves, bone vessels, bone fat, and lymph-vessels are caused by aging**. Neuropeptides, such as calcitonin gene-related peptides and vasoactive intestinal peptides, promoted human osteoblast differentiation and inhibited osteoclast formation. SP stimulated osteoblast differentiation and bone matrix mineralization, and induced BMSCs proliferation. The H-type blood vessel density of bone vessels decreased, and the vascular structure was damaged. The increase of PPARγ, miR-188, glycoprotein, and synthin-3 led to the differentiation of BMSCs into adipocytes and a large increase in bone marrow fat. The increase of P16 and P27 and the decrease of Ki67 and Vegfr3 resulted in the inhibition of lymphocyte proliferation.

The lymphatic vascular system is required to maintain interstitial fluid balance [323]. Lymphatic vessels absorb intestinal lipids, transport immune cells, and return fluid and macromolecules to the vascular system [324, 325]. Previous studies [326, 327] have found lymphatic vessels in the bones of patients with Gorham-Stout disease (GSD), a sporadic disease characterized by the presence of lymphatic vessels in bones and bone loss, which is associated with VEGF-C expression in bones [328]. Although studies [329, 330] have shown that the bones of GSD patients are filled with lymphatic vessels, it is difficult to distinguish them from lymphatic vessels morphologically because the bone has an extensive vascular network, including many dilated thin-walled vessels (sinusoids), and the presence of lymphatic vessels in normal bone remains controversial [331-335]. Only recently, Biswas *et al.* [336] demonstrated that lymphatic vessels exist in bones, and drive hematopoiesis and bone regeneration. Inhibition of lymphangiogenesis or loss of lymphatic endothelial cells (LECs) can reduce HSC regeneration after bone marrow ablation, but HSCs are not affected by LEC depletion during homeostasis. In addition, lymphatics promote bone regeneration by expanding MYH11-positive pericytes. Lineage tracing of MYH11-positive cells showed that they could expand, differentiate into adipocytes in both the bone marrow and endosteum regions, and differentiate into perivascular osteoblasts in the endosteum. Blood vessels and osteoblasts in the endothelial region are critical sites for hematopoietic regeneration of HSCs [262, 337-340]. Unlike bones from young mice, aged mice did not show lymphangiectasis under genotoxic stress. LECs purified from irradiated aged mouse bones did not up-regulate the lymphatic secretion signal CXCL12. In addition, Myh11-positive cells lack expansion in irradiated aged mouse bones. Thus, the response of LECs and MYH11-positive cells to genotoxic stress changes with age. Moreover, the expressions of p16 and p27 were up-regulated, and the proliferation marker Ki67 and lymphatic endothelial marker Vegfr3 were down-regulated in LECs of aging mice [336]. These data suggest that cell-intrinsic changes during aging may contribute to the lack of response to genotoxic stress in aged mouse LECs (Fig. 6).

### 4.5 Age-related changes in cell apoptosis and autophagy

Apoptosis is a programmed cell death process found in animals [341]. With age, the apoptosis of mouse osteocytes increases significantly [62]. This increase is related to exercise because physical activity decreases with age, which to some extent leads to bone aging and, thus, bone cell apoptosis [99, 342, 343]. In addition, the aging-enhanced oxidative stress pathway is also responsible for promoting the apoptosis of bone cells in mice [344]. This process increases mitochondrial uncoupling and superoxide production (such as ROS) and promotes bone loss by activating RANKL and upregulating sclerosis [345]. Bodine PV proved that the Wnt signal is essential in controlling osteocyte apoptosis [346].

**Figure 7.**
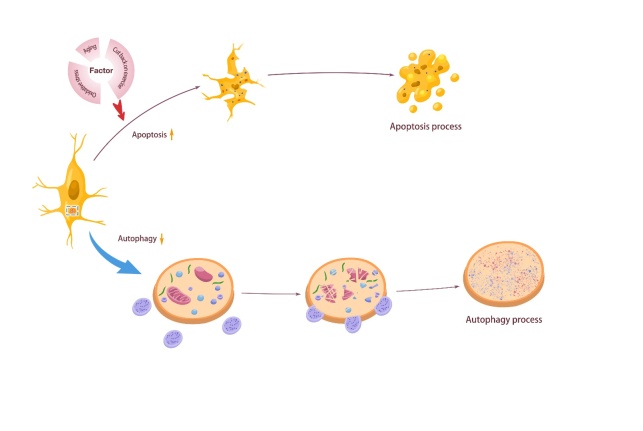
**Apoptosis and autophagy changes in bone-related cells induced by aging**. With the increase of age, the decrease of physical activity, the increase of oxidative stress pathway increased the apoptosis of osteocyte. However, autophagy of osteocyte decreased with aging.

Autophagy is a programmed cell survival mechanism [347]. As an antiapoptotic factor, it can negatively mediate cell senescence through the rapamycin signaling pathway (Mtor), thus leading to cell senescence and apoptosis [348]. Autophagy also has an essential role in the senescence of bone cells, which involves a wide range of activities. First, autophagy can partially reverse the aging of BMSCs and regulate the biological characteristics of BMSCs by affecting the levels of reactive oxygen species and p53. Compared with those in young mouse BMSCs, the autophagy and osteogenic potential in BMSCs of elderly mice are significantly reduced [349]. Liu *et al.* [350] demonstrated that the expression of optic nerve protein, which has a crucial role in selective autophagy and determines the fate of SSCs, is downregulated in aged mice. Second, autophagy participates in downregulating oxidative stress in osteoblasts and osteoblasts. In a mouse model with osteoblast-specific autophagy defects, bone loss increases significantly with aging and estrogen deficiency [351]. Autophagy protects the number of cell projections in mouse osteocytes and preserves the endoplasmic reticulum and mitochondria in osteocytes [347]. The level of autophagy in mouse osteocytes decreases with aging, and when autophagy is inhibited, bone aging can be promoted in various ways [352]. Consistent with this, LC3II/I and beclin-1, biomarkers of autophagy during autophagy vesicle nucleation, and Ulk-1, a biomarker of autophagy during autophagy formation, significantly decreases with age. In contrast, the expression of SQSTM1/p62, a protein that accumulates in autophagy, is reduced in cells, and mouse osteocyte apoptosis is increased significantly. This phenomenon shows that bone loss in elderly patients with OP may be due to the decreased autophagy ability of osteocytes but has nothing to do with apoptosis [353]. Pierrefite-Carle *et al.* [354] provided evidence that when the autophagy level of osteocytes is gradually reduced, the secretion of proinflammatory factors such as IL-1β is increased, which causes accelerated bone loss and affects bone metabolism, leading to osteoporosis. Decreased osteocyte autophagy is associated with decreased proximal tibial BMD [355]. In addition, induction of autophagy can lead to the survival response of bone marrow stromal cells to oxidative stress [356] and regulate the homeostasis of the miRNA network, which in turn regulates the homeostasis of the skeletal network [357]. The loss of autophagy may lead to increased osteocyte dysfunction in mice [358](Fig. 7).

## 5. Mechanisms of osteoimmune regulation of the bone environment in the aging state

Autoimmunity has multiple effects on the skeleton in the aging state. First, aging is closely related to immunity. Studies have shown that aging is associated with a persistent, low-grade, subclinical systemic inflammatory state[359]. However, with aging, the body appears to have "inflammatory aging," that is, an imbalance between adaptive immunity and innate immunity. Additionally, the gradual accumulation of senescent cells can occur, which can lead to increased levels of proinflammatory mediators in tissues [360]. Similarly, this inflammatory state affects bone formation and disrupts bone balance. For example, age-related systemic inflammation and the local proinflammatory environment can decrease the number and function of skeletal stem and progenitor cells (SSPCs), decrease osteogenesis, and increase adipogenesis. Inhibition of systemic inflammation can rejuvenate the function of SSPCs in aged animals, increase the number of SSPCs, and improve the osteogenic differentiation potential [361]. Studies have shown that the RELA proto-oncogene (NF-kB subunit, RELA), an essential subunit of the NF-kB complex, plays a vital role in inflammation [362]. Moreover, it is activated in bone tissues of middle-aged and older people and causes an inflammatory response in bone cells in the aging state [363]. Persistent chronic inflammation leads to increased osteoclast activation and decreased osteoblast formation, thereby increasing bone resorption and reducing bone formation during healing [9]. In addition, the generation of age-associated B cells (ABCs) in the bone marrow of aging mice is increased, and these cells secrete higher levels of TNF-α. TNF-α impairs the production of growing B cells [364]. This leads to a reduction in both the number and function of B-cell precursors in the bone marrow of aging mice [365]. Plasma cells that accumulate in the bone marrow of aged mice can generate a feedback loop of proinflammatory cytokines, such as IL-1 and TNF-α, which promote the differentiation of HSCs to myeloid cells. This leads to a decrease in the production of hematopoietic stem cells into lymphoid cells, impairs immunity [366], and further aggravates bone aging. Moreover, when inflammation is excessive, both acute and chronic, it favors adipogenic differentiation [367].

The regulatory mechanism of autoimmunity in bone aging is mainly achieved by the secretion of proinflammatory factors by the immune system in the aging state. Bone marrow cells of aging mice secrete more IL-1α/β ^[15]^. Similarly, Salvioli et al. [368] also found that the expression levels of TNF-α, IL-1, IL-6, and other cytokines increased with age. IL-1α/β, IL-6 and IL-8 are proinflammatory factors that can activate SASP [369-371]. SASP is one of the markers of many senescent cells [368] and represents the low-grade chronic inflammatory state of the body with aging [372]. Interestingly, the proinflammatory phenotype of SASP is mediated by amplified signaling in the NF-κB cascade [373]. Similarly, aging-induced SSPC changes were found to be associated with NF-κB activation. Inhibition of NF-κB activation can restore the function of aging SSPCs, improve the symptoms of aging, increase the number of SSPCs, and enhance osteogenic function [361]. The increase in IL-6 and the decrease in IL-10 in the inflammatory microenvironment reduced the stemness of mouse BMSCs through the JAK-STAT signaling pathway, resulting in weakened osteogenic differentiation [374]. In addition, IFN-γ and TNF levels are increased in the bone marrow of aged mice [375] to regulate bone balance. IFN-γ can both inhibit and promote osteoclast formation, depending on the stage of osteoclast precursor at the time of IFN-γ addition and the concentration of IFN-γ [376]. In conclusion, the effects of IFN-γ on osteoclasts are complex. In addition, IFN-γ can promote HMSC differentiation into osteoblasts [377]. The proportion of Th17 cells and the expression levels of IL-17A and IL-6 in the spleen of mice at different ages increased with age [378]. IL-17 has a dual effect on bone metabolism, depending on the cytokine milieu and the bone site [379]. First, IL-17 is an osteoclast-promoting cytokine that is mainly derived from Th17 cells [380]. IL-17 inhibits the expression of Dkk1, an inhibitor of the Wnt system, at the peripheral level [381] to regulate the RANKL/OPG ratio [382], and this inhibition is enhanced under low TNF-α conditions [381]. In addition, Russel et al. [383] confirmed that IL-17 directly promoted the differentiation of MSCs into mature osteoblasts. Therefore, osteoimmunity can affect bone metabolism by regulating IL-17 secretion. In conclusion, we found that the osteoimmune system participates in regulating bone metabolism in the aging state mainly through the secretion of inflammatory factors, and multiple signaling pathways are involved.

## 6. Treatment for osteoporosis caused by aging

Remodeling continues throughout life. When total bone formation and resorption are equal, BMD is stable [384]. However, this balance gradually breaks with age, leading to OP and bone loss. OP is a systemic bone disease characterized by osteopenia and fracture susceptibility and a significant bone metabolic disease closely related to aging [385]. With the acceleration of population aging, the incidence of OP has been gradually increasing. The National Osteoporosis Foundation reported in 2016 that one in two women and one in four men over 50 have a bone would fracture due to OP. Also, some studies predicted that the number of OP patients in China would reach 212 million by 2050, thus causing which will cause a substantial social and economic burden. Therefore, prevention and treatment of OP have become urgent public health problems to be solved [386, 387].

### 6.1 Drug therapy

According to the discovery methods of related drugs, the current findings that support the treatment of osteoporosis can be divided into multiple categories. First, they are discovered through clinical observation, including the levels of estrogen, parathyroid hormone, and parathyroid hormone-related peptide analogs. Second, they are discovered through medical chemical or physiological research, such as calcitonin and selective estrogen levels. Third, some are discovered by chance, such as bisphosphonates (water softeners), zoledronate, and alendronate sodium levels. Finally, they are discovered by advanced molecular observations, including the levels of apatite, denosumab (RANKL antibody), and roosozumab (sclerostin antibody) [388]. Bisphosphonates, including alendronate, risedronate, ibandronate, and zoledronate, are the most used anti-absorptive drugs for the treatment of osteoporosis. The mechanism of action of bisphosphonates is to inhibit farnesyl diphosphate synthase in the mevalonate pathway and to prevent the isolation of GTP binding proteins, which are essential in the function and metabolism of osteoclasts in the cellular skeleton. This class of drugs can inhibit osteoclast activity and bone turnover, increase bone mineral density, reduce fractures, and improve the survival rate of OP patients [389]. Bisphosphonates, especially alendronate and risedronate, can effectively improve bone mineral density [390]and are the only two therapies with a significant combined treatment effect on non-vertebral fracture reduction [391]. Treatment with zoledronic acid for five years from early menopause reduces the risk of long-term fracture and the proportion of older women with femoral neck BMD OP [392]. Bortezomib is a novel anabolic of bisphosphonates used to treat postmenopausal and age-related bone loss [393]. Yet, bisphosphonates often have side effects. However, flavonoid-induced autophagy has a cytoprotective effect against zoledronate toxicity while supporting osteogenic transcription factors, promoting osteoblast differentiation and bone formation [394]. Isoflavones, a subclass of flavonoids, are structurally similar to estrogen and can be used as hormone replacement therapy to inhibit osteoclast differentiation and prevent postmenopausal OP [395]. Teriparatide or alternatives, such as strontium, may be considered in cases where bisphosphonates are unsuitable or ineffective. Teriparatide treatment significantly reduces the risk of vertebral and non-vertebral fractures, and back pain, and improves the quality of life [396]. In addition, strontium ranelate can stimulate bone formation and inhibit bone resorption [397], and preclinical data support that strontium ranelate implants improve fracture healing and osseointegration [398]. In preclinical and short-term clinical studies, lasofoxifene has shown certain efficacy in preventing bone loss and reducing cholesterol levels. Also, 0.25 mg/day of lasofoxifene was used as the lowest fully effective dose in the dose model of the phase II study [399].

In recent years, some new drugs have gradually entered the field of osteoporosis treatment. For example, 17b-HSD2 inhibitors can maintain high levels of estradiol and testosterone in bone and protect against bone loss [400]. The compound hydroxyphenyl keto-thiophene has been identified as a moderate 17b-HSD2 inhibitor (IC50 of 382 nM) [401]. In addition, inhibitory L-plastin peptide can block osteoclast function without damaging osteoblast function and can be developed as a prospective therapeutic agent for the treatment of OP [402].

Moreover, traditional Chinese medicine has a vital role in preventing osteoporosis. Eumoides [403], Epimedium [404], and Liuwei Dihuang pill [405], a classic formula for invigorating kidneys, have been widely used in the treatment of postmenopausal OP for a long time. Qing'e-decoction has also been confirmed to regulate bone metabolism, improve bone quality and delay bone aging, which may be achieved by increasing sex hormones, serum leptin receptor, and leptin receptor [406]. Pharmacological studies have shown that Kidney tonifying in traditional Chinese medicine can promotes osteoblasts, inhibits osteoclasts, regulates estrogen levels, etc; promote osteogenesis, inhibit bone marrow mesenchymal stem cell adipogenesis, regulate calcium and phosphorus metabolism, and inhibit oxidative stress. These effects are mediated by the OPG/RANKL/RANK, BMP/Smads, MAPK, and Wnt/β-catenin systems [407]. Salidroside can prevent OP by inhibiting oxidative stress, up-regulating Nrf2 to promote osteogenesis, and interfering with galactose and fatty acid metabolism [408].

### 6.2 Exercise

Drug therapy is effective in delaying bone loss in the elderly; however, there are economic pressures and potentially toxic side effects of long-term medication. On the other hand, proper exercise physical activity can promote bone formation and delay bone mass loss caused by aging. Therefore, the World Health Organization also considers exercising one of the primary non-drug therapies for preventing and treating OP [409]. In addition, exercise can help prevent and treat osteoporosis with relatively few side effects.

Endurance exercise training of moderate and above intensity effectively improves cardiopulmonary function, prevents and treats chronic metabolic diseases, and regulates the psychological and mental state. Stones *et al.* [410] confirmed that aerobic endurance and resistance exercises could positively benefit bone metabolism. However, this effect is weakened with age. Resistance exercise has been widely used in treating OP and muscle atrophy caused by various reasons and has achieved remarkable results. Nelson *et al.* [411]demonstrated the effectiveness of high-intensity strength training in maintaining femoral neck BMD and improving muscle mass, strength, and balance in postmenopausal women. The most effective type of exercise intervention for femoral neck BMD is progressive resistance training of the lower limbs. In contrast, the BMD of the spine is improved by weight-bearing and resistance training [412]. Moreover, sprint, football, basketball, and other sports are famous for their fast rhythm, fierce confrontation, and strong competitiveness, which require higher physical functions of participants and have a greater probability of sports injuries. However, speed and impact exercises provide excellent mechanical stimulation to the bone, which is also considered practical bone-strengthening exercises. Suominen *et al.* [413] evaluated the effect of continued strength and sprint training on bone aging in 40-85 year-old male sprinters with a long-term training background, finding that continuous sprint and strength training can maintain or even improve the tibial characteristics of middle-aged and elderly male sprinters. Flexibility and balance exercises are primarily aerobic exercises, such as gymnastics, yoga, pilates, and traditional Chinese exercises, such as Tai Chi and Baduanjin, have also been recognized as suitable types of exercises that can strengthen the flexibility of soft tissues and improve the balance and coordination of the body. These interventions are also widely spread around the world. Tai chi can benefit patients by improving BMD value and bone Gla protein level and relieving osteoporosis pain [414].

### 6.3 Stem Cell Transplantation

Stem cell transplantation has shown great potential in the treatment of osteoporosis. BMSCs have the characteristics of easy access and high differentiation potential. Through partial transplantation, the thickness of bone trabeculae can be increased, the newly formed bone with microstructure can be improved, and the hardness of bone can be increased [415]. ADSCs, administered intravenously, can prevent bone loss, improve trabecular bone quality, and increase the expression of molecular markers related to bone turnover in ovariectomy-induced, age-related, and other osteoporosis models [415]. In addition, dental pulp stem cells can mediate tissue regeneration and promote the expression of osteogenesis-related genes through systemic infusion [416, 417]. UCMSCs, when partially injected, can enhance osteoblast differentiation, increase trabecular bone formation, and reduce bone loss [418, 419].

Vesicles derived from normal skeletal muscle can be phagocytosed by bone marrow stromal/stem cells and osteoclasts and promote osteogenic differentiation of BMSCs while inhibiting osteoclast formation as a cell-free therapy to treat disuse OP [420].

**Table 4:** Treatment for osteoporosis caused by aging.

Treatment for osteoporosis	Specific treatment measures
**Drug therapy**	The drugs for the prevention and treatment of OP could be generally divided into bone resorption inhibitors, including calcium, estrogen, calcitonin, bisphosphonates, vitamin D, and bone formation promoters, including fluoride, parathyroid hormone, vitamin K2, and growth factors. Also, some drugs had the above two bidirectional regulatory effects such as estrogen, calcitonin, vitamin D, and statins. Traditional Chinese medicine involved eumoides, epimedium, liuwei dihuang pill, qing'e-decoction and salidroside.
**Exercise**	Resistance exercise, strength training, high intensity exercise, flexibility and balance exercise.
**Stem cell transplantation**	ADSCs, dental pulp stem cells, UCMSCs, and vesicles derived from normal skeletal muscle.
**Bioactive materials**	3D printing biomimetic scaffolds and nanomaterials.
**Nutritional and hormonal supplements**	Vitamin D3.

### 6.4 Bioactive materials

Bioactive materials have also shown great potential in the treatment of OP. Montalbano *et al.* [421] developed 3D-printed biomimetic scaffolds with hybrid bioactive materials consisting of type I collagen and strontium-containing mesoporous bioactive glasses to promote osteogenesis. Similarly, Main *et al.* [422]invented a customized implant composed of mineralized collagen to repair massive weight-bearing bone defects. Moreover, nanomaterials have also been found to be effective for bone repair [423]. Graphene, silicate nanoparticles, and layered double hydroxides are emerging as strong competitors for bioactive nanomaterials for new bone formation in the next generation of bone tissue engineering and OP therapy [424]. Other inorganic nanomaterials, such as silica nanoparticles and nano-hydroxyapatite, have been shown to induce osteogenic differentiation of stem cells and promote new bone formation [425]. Nano-hydroxyapatite can stimulate the osteogenic differentiation of stem cells and promote the formation and mineralization of new bone tissue [424].

### 6.5 Nutritional and hormonal supplementation

The effects of macronutrients, such as protein, lipids, and carbohydrates; micronutrients, such as minerals calcium, phosphorus, and magnesium, as well as vitamins D, C, and K; flavonoid polyphenols, such as quercetin, rutin, luteolin, kaempferol, and naringin, are essential for the prevention and treatment of OP [426]. Adequate vitamin D is essential to maximize bone health by promoting intestinal absorption of calcium and phosphorus. Patients undergoing OP therapy should be appropriately supplemented with calcium and vitamin D to maximize the therapeutic effect [427]. According to the Institute of Medicine, a calcium intake of 1200 mg per day is recommended for postmenopausal women with an upper limit of 2000 mg, preferentially obtained from the diet [428].

Melatonin has the potential as a novel anti-OP therapy. It mainly acts through its cognate receptors, in which melatonin receptor two is expressed in BMSCs, osteoblastic bone formation, and osteoclast bone resorption. Melatonin favors the osteoblast fate of SSC, stimulates the survival and differentiation of osteoblasts, and inhibits the osteoclast differentiation of hematopoietic stem cells [429].

**Figure 8.**
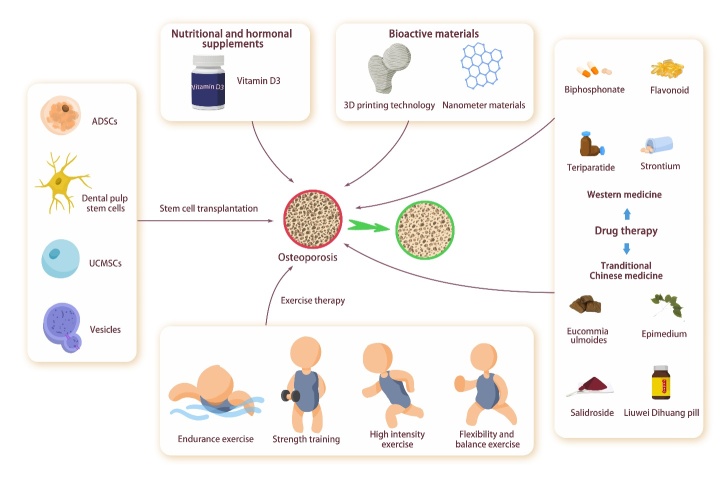
**Treatment of osteoporosis caused by aging**. The treatment of osteoporosis mainly involved the following aspects: through drug treatment, such as bisphosphonates, flavonoids, teriparatide, strontium, eucommia ulmoides, epimedium, liuwei dihuang pills, salidroside, etc. Exercise therapy, such as resistance exercise, strength training, high intensity exercise, flexibility, and balance exercise. Stem cell transplantation treatments, such as ADSCs, dental pulp stem cells, UCMSCs, and vesicles derived from normal skeletal muscle. The treatment of bioactive materials involved 3D printing biomimetic scaffolds and nanometer materials. Treatment also included nutritional and hormonal supplements, such as vitamin D3.

Alginate oligosaccharide has anti-OP activity in mice with OP, which can inhibit osteoclast proliferation, promote osteoprotegerin expression, competitively inhibit the binding between RANK and RANKL in senile OP, and reduce serum osteocalcin secretion and bone transformation [430]. The water extract of Boseokchal can potentially treat osteoporosis by inhibiting RANKL-induced osteoclastogenesis in a dose-dependent manner and reducing the resorption activity of osteoclasts without producing a cytotoxic effect on bone marrow-derived macrophage cells [431] (Fig. 8).

## 7. Summary

With aging, the incidence of skeletal system diseases has been increasing. Therefore, a comprehensive understanding of the changes in bone caused by aging is essential for the prevention and treatment of skeletal-related diseases. With aging, the microenvironment within bone favors bone resorption and inhibits bone formation, and this is accompanied by bone marrow fat accumulation and multicellular senescence. Specifically, BMSCs tend to undergo adipogenic rather than osteogenic differentiation during aging, which is accompanied by a decrease in BMSC number and a partial functional decline. Osteoblasts and osteocytes exhibit increased apoptosis, decreased numbers, and multiple functional limitations, leading to attenuated bone formation. In addition, the increased activity of osteoclasts enhances bone resorption. Senescent cells exhibit changes in the composition of the cell membrane, the substances in the cytoplasm, the morphology and function of the nucleus and mitochondria, the activity of lysosomes, and the substances and functions secreted by exosomes.

The bone microenvironment in the aging state is mainly reflected in the reduction and functional impairment of type H vessels in the bone vascular system, a significant increase in adipose tissue and even infiltration in bone, weakened bone nerve function, and reduced bone lymphocytes. By reviewing the relevant literature, we found that the bone microenvironment shows unlimited potential for targets for clinical therapies of skeletal system diseases caused by aging. In addition, the current studies are often scattered and superficial, show weak targets, and lack holistic studies on the complete bone microenvironment. Most current research is still in the animal and cell experiment stages. Studies on bone-related cells affected by aging have focused on whole cells and paid less attention to cellular organelles and expression products. Hence, basic research on the bone microenvironment needs to be further developed to provide a solid foundation for subsequent transformation into clinical applications.
